# Familial Mediterranean Fever Mimicking Whipple’s Disease: A Case Report and Review of the Literature

**DOI:** 10.31138/mjr.33.2.252

**Published:** 2022-06-30

**Authors:** Sofia Flouda, Georgia-Savvina Moysidou, Christina Damoulari, Noemin Kapsala, Maria Kosmetatou, Anastasia Antoniadou, Dimitros Boumpas, Pelagia Katsimpri

**Affiliations:** 1 4th Department of Internal Medicine, Attikon University General Hospital, Medical School, National and Kapodistrian University of Athens, Athens, Greece; 2 Biomedical Research Foundation Academy of Athens

**Keywords:** familial Mediterranean fever, Whipple’s Disease

## CASE REPORT

A 60-year-old male was admitted to our hospital because of fever of 38°C, chronic diarrhoea (sometimes bloody), fatigue, and weight loss (12kg the last 6months).

His past medical history included a diagnosis of FMF a year ago (based on recurrent episodes of fever lasting 4days, migratory arthritis, and a recurring laboratory inflammatory syndrome during the last 10years and heterozygosity for the P369S mutation of the MEFV), IgA kidney disease biopsy proven, hypothyroidism, dyslipidaemia and gastroduodenitis due to H. pylori.

His current treatment included canakinumab 150mg/4wks (began in the last month due to intolerance to previous treatment with colchicine), atorvastatin, allopurinol, T4 and β-blocker.

Physical examination revealed cachexia, symmetric inflammatory polyarthritis of metatarsophalangeal joints and swollen lower extremities with pitting oedema.

**Figure 1.**
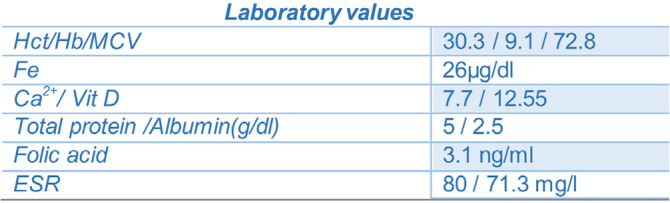
Laboratory evaluation of the reported patient with a diagnosis of FMF (heterozygous of P369S) under treatment with canakinumab and FUO, chronic diarrhoea, and malabsorption. Electrocardiogram revealed no abnormalities.

Initial complete blood cell count and biochemical tests revealed: hypochromic microcytic anaemia (haemoglobin: 9.1 g/dL, haematocrit: 30.3 %, MCV: 72.8%) and malabsorption with iron and folic deficiency (Fe: 26μg/dl- Folic acid: 3.1ng/ml), hypocalcaemia corrected for albumin (Ca2+: 7.7mg/dl, NR: 8.4–10.2mg/dl), hypoalbuminemia (Albumin: 2.5g/dl, NR: 3.5–5.5g/dl), low levels of Vitamin D (17.55ng/ml) and raised C-reactive protein:71.3 (NR: 0–5) mg/dL and ESR: 80 (**Figure 1**). Stool testing did not reveal any pathogenic microorganisms.

**Figure 2.**
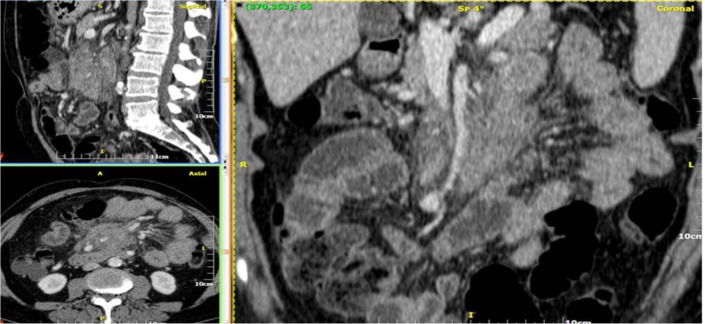
CT scan of the abdomen: paraaortic and mesenteric lymphadenopathy, hyperaemia/congestion of the mesenteric vessels, turbidity of the mesenteric fat, and enteritis.

The patient underwent work up for fever of unknown origin (FUO) with chest and abdominal CT scan. CT scans and endoscopy of his gastrointestinal tract as work up for FUO had been carried out prior to the diagnosis of FMF and was unremarkable. This time CTs revealed paraaortic and mesenteric lymphadenopathy, hyperaemia and congestion of the mesenteric vessels, turbidity of the mesenteric fat and enteritis (**Figure 2**).

**Figure 3.**
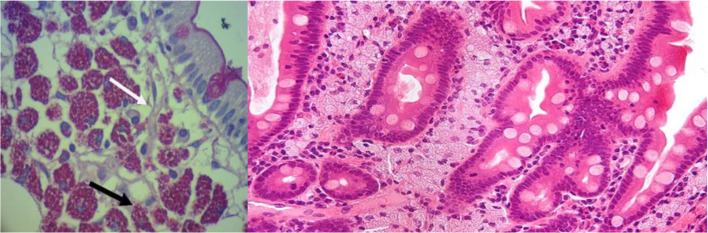
Periodic Acid-Schiff (PAS) staining-Marked accumulation of PAS-positive macrophages (white arrow) on submucosa -Presence of fibrosis (black arrow) suggesting a chronic process- Rod-shaped microorganisms - typically in macrophages- Lamina propria macrophages usually abundant.

Repeat endoscopy and biopsies of the upper and lower gastrointestinal tract revealed lymphatic hyperplasia of the duodenum and ileum. PAS staining was positive for Tropheryma whipplei. PCR was, also, positive for Whipple’s disease (**Figure 3**).

Lumbar puncture and PCR of the CSF was performed to record the extent of the Whipple’s disease which was negative for Tropheryma whipplei. Canakinumab was stopped and the patient received iv ceftriaxone 2gr daily for 2weeks followed by co-trimoxazole three times daily for maintenance therapy for 12months. Two weeks later, he was afebrile, he had gained weight, and the laboratory findings of malabsorption were restored to within normal range. However, his renal function deteriorated due to toxicity of co-trimoxazole therefore maintenance therapy for Whipple’s disease was switched to a combination of doxycycline 100mg twice daily and hydroxychloroquine 200mg three times daily. During follow-up the renal function returned to normal, and the patient continued to improve.

## DISCUSSION

Whipple’s disease (WD) is a chronic systemic bacterial infection caused by Tropheryma whipplei, a ubiquitous commensal bacterium. It affects mostly middle-aged men and is more frequent among sewer workers.^1^ In the general population, the prevalence of healthy carriers (positively detected and identified by PCR of the bacterium in stools) is up to 7%.^2^ Transmission is mostly faecal-oral, but its detection in the saliva suggests also an oral-oral transmission.^3^

The classic form of WD presents with recurrent, intermittent arthritis (affecting mostly large joints) followed by diarrhoea and malabsorption, leading to weight loss. However, a broad spectrum of symptoms has been observed including unexplained prolonged fever, blood culture negative endocarditis, central nervous system or pulmonary involvement, uveitis, and spondylodiscitis.^2^

When suspecting WD the finding of Tropheryma whipplei by PCR in saliva, stools and/or synovial fluid in the presence of arthritis is necessary, and in the case of positive testing, biopsy of the small intestine should be performed. PAS positive staining confirms the diagnosis. A PCR analysis of the CSF before treatment is also recommended.

Untreated, Whipple’s disease can be fatal. The treatment typically recommended is oral trimethoprim-sulfamethoxazole twice daily for 1–2 years preceded by a parenteral administration of Ceftriaxone for 2 weeks. Corticosteroid and immunosuppressant drugs are harmful.

Atypical presentations of WD may lead to misdiagnosis as it can mimic other infectious, autoimmune, granulomatous, or inflammatory diseases,^4,16^ as was the case in our patient.

In the literature there are many case reports of patients with an initial diagnosis of rheumatic disease that turned out to be WD during follow up,^10–14^ as well as cases of WD exacerbation after treatment with a biologic agent.^15^ Quartuccio et al. describe 2 patients originally diagnosed as seronegative arthritis who received several years treatment with both cDMARDs and bDMARDs before the diagnosis of WD. Makol et al. describe a case of pericarditis that even received rituximab as per severe rheumatoid pericarditis before the diagnosis of WD.^12^ Ramos et al. describe 2 patients, a 58-year-old with psoriatic spondyloarthritis who received 5 different bDMARDs and a 73-year-old with a diagnosis of rheumatoid arthritis who received 3 different bDMARDs before the diagnosis of WD.^15^

Familial Mediterranean fever (FMF) is the most common monogenic, autosomal recessive autoinflammatory disease associating unprovoked recurrent febrile episodes with serositis and arthritis.^5^ Its diagnosis is currently based on the Tel-Hashomer clinical criteria, in which two or more major symptoms (including recurrent febrile episodes with serositis, amyloidosis of AA type without a predisposing disease and favourable response to regular colchicine treatment) or one major plus two minor symptom (including recurrent febrile episodes, erysipelas-like erythema and FMF in a first-degree relative) are needed.^19^

Molecular genetic confirmation is not essential for diagnosis but can be used as an adjunct in atypical cases. The marenostrin-encoding fever (MEFV) gene is the only gene associated with FMF and is located on chromosome 16p13, with exons 10, 3, and 2 of MEFV being the major sites of disease-causing mutations.

In patients with atypical attacks and one mutation (heterozygotes), genetic test cannot exclude or confirm FMF as a possible diagnosis.^8^ The P369S mutation, present in our patient is one of the described mutations,^6^ on exon 10,^23^ found in 3.3% of Turkish patients with a diagnosis of FMF.^7^ In another study the P369S mutation was seen in 3.5% of heterozygous patients, 0.2% of compound heterozygous patients, 0.5% of homozygous patients and 61.8% of patients with complex alleles.^21^ This mutation is associated with a high frequency of fever and abdominal pain and less frequently chest pain and arthritis. Genetic diagnosis is definite when two mutations, identical (homozygous) or nonidentical (complex heterozygous), are present in the two MEFV alleles; if only one or no pathogenic mutation is found the diagnosis of FMF based on clinical criteria is still possible due to the potential occurrence of novel mutations.^20^

Presentation of FMF is usually before the age of 20 but late onset FMF is described and represents a distinct, milder form of the disease.^22^

Colchicine remains the corner stone in the treatment of FMF and it can also be used to confirm the diagnosis in atypical cases. Interleukine-1 (IL-1) inhibitors can be used in case of intolerance or insufficient response to treatment.^9^ Our patient, heterozygous for the P369S mutation, presenting with fever, abdominal pain and arthritis fits the clinical presentation for FMF. However, patient’s partial response, the chronic diarrhoea and malabsorption and intolerance to colchicine go against the diagnosis of FMF.

In summary, Whipple’s disease is a rare systemic infectious disease with a frequently heterogeneous and atypical presentation capable of mimicking other infectious or inflammatory diseases. FMF may also be missed especially when presenting in an atypical fashion and here gene testing may be helpful if the result is homozygosity or compound heterozygosity. However, care should be taken in applying the diagnosis in heterozygous cases. Thus, a high suspicion for Whipple disease’s is needed for timely diagnosis and targeted treatment in order to avoid the potentially lethal complications of the disease.

Immunosuppressive drugs may aggravate the symptomatology and should be avoided in Whipple’s disease. Patients with seronegative arthritis, abdominal symptoms and insufficient response to treatment should be screened for Whipple’s disease.
